# Poor sleep quality and loneliness among higher vocational college students: a conditional process analysis of psychological resilience and self-esteem

**DOI:** 10.3389/fpsyg.2026.1893857

**Published:** 2026-08-21

**Authors:** Lan Shi, Yanjuan Zhou, Peipei Cai

**Affiliations:** 1 School of Engineering and Technology, Harbin Vocational College of Science and Technology, Harbin, Heilongjiang, China; 2 School of Marxism, Harbin Medical University, Harbin, Heilongjiang, China; 3 Department of Accounting and Finance, Heilongjiang Polytechnic, Harbin, Heilongjiang, China

**Keywords:** higher vocational college students, loneliness, moderated mediation, psychological resilience, self-esteem, sleep quality

## Abstract

**Background:**

Loneliness, sleep quality, psychological resilience, and self-esteem are closely related to young adults’ everyday psychological adjustment. However, less is known about how sleep quality is associated with loneliness among higher vocational college students and whether this association depends on internal psychological resources. This study examined the association between sleep quality and loneliness and tested the mediating role of psychological resilience and the moderating role of self-esteem.

**Methods:**

A cross-sectional survey was conducted from May to December 2023 in three higher vocational colleges in Heilongjiang Province, China. A total of 4,000 questionnaires were distributed, and 3,782 valid responses were included in the final analysis. Sleep quality, psychological resilience, self-esteem, and loneliness were assessed using the Pittsburgh Sleep Quality Index, 10-item Connor-Davidson Resilience Scale, Rosenberg Self-Esteem Scale, and 20-item UCLA Loneliness Scale, respectively. Pearson correlation analysis, multiple linear regression analyses, bootstrap mediation analysis, and moderated mediation analysis were performed. Fully adjusted models controlled for age, sex, grade, residence, family economic status, parental marital status, dormitory relationship, classmate relationship, academic pressure, and physical activity frequency.

**Results:**

Among the 3,782 participants, the mean age was 19.35 years (SD = 1.31); 1,593 participants were male (42.1%) and 2,189 were female (57.9%). Sleep quality was positively correlated with loneliness and negatively correlated with psychological resilience and self-esteem. Psychological resilience and self-esteem were negatively correlated with loneliness. In the fully adjusted mediation model, poorer sleep quality was associated with higher loneliness (*β* = 0.556, *p* < 0.01) and lower psychological resilience (*β* = −0.491, *p* < 0.01). Psychological resilience partially mediated the association between sleep quality and loneliness, with an indirect effect of 0.701, accounting for 45.93% of the total effect. The interaction between psychological resilience and self-esteem was significant (*β* = 0.119, *p* < 0.01). Conditional indirect effects were strongest among students with low self-esteem and weakest among those with high self-esteem, and the index of moderated mediation was significant.

**Conclusion:**

Poorer sleep quality was associated with higher loneliness among higher vocational college students, and this association was partly explained by lower psychological resilience. Self-esteem further buffered the psychological resilience–loneliness pathway. These findings suggest that combining sleep health education, resilience enhancement, and self-esteem support may provide useful directions for reducing loneliness among higher vocational college students.

## 1 Introduction

Loneliness is a subjective and distressing experience that arises when individuals perceive a discrepancy between their desired and actual social relationships (Baumeister and Leary, 1995). It differs from objective social isolation because it reflects the perceived quality, adequacy, and emotional meaning of social connection (Wodika et al., 2025). The need to belong has long been considered a fundamental human motivation, and insufficient fulfillment of this need may undermine emotional regulation, cognitive appraisal, interpersonal functioning, and well-being (Hawkley and Cacioppo, 2010). In recent years, loneliness has attracted growing attention as a psychological and social health issue among adolescents and young adults (Shah and Househ, 2023; Twenge et al., 2021). Evidence from cross-national and developmental research suggests that loneliness has increased among young people and is particularly relevant during emerging adulthood, a period characterized by identity exploration, residential transition, peer relationship reconstruction, and increasing independence (Hernández-Díaz et al., 2025; Lo et al., 2026). For students in higher vocational education, these developmental tasks may be further intertwined with academic pressure, dormitory living, peer adaptation, and early preparation for employment (Jain et al., 2026). Compared with students in traditional universities, higher vocational college students typically receive more practice-oriented and occupation-focused training and may face employment preparation at an earlier stage of their education. These distinctive educational and developmental circumstances may shape their sleep patterns, psychological resources, and interpersonal experiences. Nevertheless, this population remains relatively underrepresented in research on loneliness and its psychological correlates.

Sleep quality may represent an important everyday vulnerability factor associated with loneliness (Ben Simon and Walker, 2018). Sleep is not only a biological recovery process but also a foundation for emotional regulation, cognitive control, stress tolerance, and interpersonal sensitivity. Poor sleep quality may increase fatigue, irritability, negative affect, and vigilance toward social threat cues, making ordinary interpersonal difficulties more likely to be interpreted as rejection, exclusion, or disconnection. A systematic review and meta-analysis showed that loneliness is associated with sleep disturbance, particularly self-reported sleep quality and insomnia symptoms, while the evidence regarding sleep duration is less consistent (Griffin et al., 2020). Recent studies among college students have also reported close associations between loneliness and sleep quality and have begun to examine psychosocial mechanisms linking these constructs (Dong et al., 2025). The relationship between sleep-related difficulties and interpersonal functioning may also operate within a broader social context. For example, recent evidence among adolescents showed that social connectedness was associated with fewer sleep problems and mediated the association between family conflict and sleep-related difficulties, indicating that sleep and perceived social connection may be closely intertwined (Çi̇çek and Yıldırım, 2025). At the same time, sleep quality is a potentially modifiable health-related condition. Recent evidence suggests that psychological interventions can improve sleep quality among young adult university students, indicating that sleep problems may be amenable to intervention in educational settings (Tadros et al., 2025). Therefore, examining the association between sleep quality and loneliness may help identify an everyday and potentially modifiable correlate of loneliness among higher vocational college students.

Psychological resilience may help explain the association between sleep quality and loneliness. Resilience refers to the capacity to maintain or regain adaptive functioning when facing stress, adversity, or developmental challenges (Sullivan et al., 2025). Conservation of resources theory provides a useful framework for understanding this pathway: people seek to obtain, preserve, and restore valued psychological resources, and stress is more likely to occur when these resources are threatened, lost, or insufficiently replenished (Hobfoll, 1989). Within this framework, adequate sleep may support the restoration of emotional and cognitive resources, whereas persistent poor sleep quality may interfere with recovery and contribute to progressive resource depletion. Students experiencing such depletion may have fewer psychological resources available to cope with academic demands, dormitory conflicts, interpersonal uncertainty, and concerns about future employment. Poor sleep quality may therefore undermine recovery and reduce the psychological resources needed for coping with academic and interpersonal demands. Empirical studies have shown that sleep quality is associated with psychological resilience among college students, and that poorer sleep quality may be linked to lower resilience (Zhang et al., 2025). In addition, resilience has been identified as a protective factor against emotional and social loneliness among college students (Labrague et al., 2021). Recent research among university students has similarly reported that loneliness is associated with lower resilience and identified resilience as a psychological mechanism involved in the relationship between loneliness and problematic social media use (Çiçek et al., 2026). Students with lower resilience may have greater difficulty recovering from academic stress, dormitory conflicts, and interpersonal uncertainty, thereby experiencing stronger feelings of social disconnection and loneliness. Based on this logic, psychological resilience may operate as a mediating mechanism linking poor sleep quality to loneliness. Accordingly, Conservation of Resources Theory suggests that reduced resilience may represent one mechanism through which inadequate recovery associated with poor sleep quality is linked to greater loneliness.

Self-esteem may further shape the pathway from psychological resilience to loneliness. Self-esteem reflects an individual’s overall evaluation of self-worth and self-acceptance. Sociometer theory proposes that self-esteem functions as a psychological monitor of perceived relational value, indicating the extent to which individuals feel accepted, valued, or rejected by others (Peng et al., 2019). From this theoretical perspective, fluctuations in self-esteem reflect individuals’ perceptions of their relational standing and sensitivity to potential social rejection. Self-esteem is closely related to perceived relational value and social belonging. Students with higher self-esteem may preserve a more stable sense of self-worth even when experiencing stress or interpersonal difficulties, whereas those with lower self-esteem may be more likely to interpret setbacks as signs of inadequacy, rejection, or exclusion. Empirical evidence among university students has shown that loneliness is negatively associated with self-esteem and that self-esteem is linked to psychological and subjective well-being (Çiçek, 2021). Self-esteem is also closely associated with adaptive psychological resources. Evidence among university students suggests that self-esteem may help explain how positive developmental experiences are related to psychological resilience (Kocatürk and Çiçek, 2023). In addition, recent findings indicate that lower self-esteem is associated with greater loneliness and may partly explain the relationship between problematic social media use and loneliness among university students (Ünsal et al., 2025). Other recent work has also indicated that self-esteem may moderate the relationship between loneliness and depressive symptoms among university students (Ho, 2024). Therefore, self-esteem may serve as a boundary condition in the association between psychological resilience and loneliness. Specifically, lower self-esteem may strengthen the link between reduced resilience and loneliness, whereas higher self-esteem may buffer this association. Students with higher self-esteem may retain a relatively stable sense of personal and relational value despite reduced resilience, while students with lower self-esteem may be more likely to interpret limited coping capacity as evidence of social inadequacy or rejection.

Despite increasing interest in loneliness among young people and college students, several issues remain insufficiently addressed. Higher vocational college students have received relatively limited empirical attention, although their educational experience is shaped by dormitory living, skill-oriented training, and early preparation for employment (Chen, 2024). Existing studies have more often examined loneliness in relation to social support, belonging, or general mental health problems, whereas the role of everyday health-related vulnerability, such as poor sleep quality, remains less clear. Although resilience and self-esteem have each been linked to loneliness, they have commonly been examined as separate protective factors rather than as components of an integrated explanatory model. Consequently, limited evidence is available regarding whether resilience explains the association between sleep quality and loneliness and whether this indirect association varies according to students’ self-esteem. The present study therefore addresses three related gaps: the limited evidence concerning higher vocational college students, the insufficient examination of sleep quality as an everyday and potentially modifiable correlate of loneliness, and the lack of an integrated model incorporating both an explanatory mechanism and a psychological boundary condition.

Taken together, Conservation of Resources Theory and Sociometer Theory provide complementary explanations for the proposed conditional process model. Conservation of Resources Theory explains how poor sleep quality may be associated with loneliness through inadequate resource restoration and reduced psychological resilience. Sociometer Theory explains why the association between resilience and loneliness may differ according to students’ self-esteem and perceived relational value. In the present model, psychological resilience was therefore conceptualized as a potential mediating mechanism linking sleep quality to loneliness, whereas self-esteem was conceptualized as a moderating condition that may alter the strength of the resilience–loneliness association. To address these gaps, the present study examined the association between sleep quality and loneliness among higher vocational college students and tested a conditional process model involving psychological resilience and self-esteem.

Based on the theoretical and empirical evidence above, the following hypotheses were proposed:

H1: Poorer sleep quality will be positively associated with loneliness among higher vocational college students.

H2: Psychological resilience will mediate the association between sleep quality and loneliness. Specifically, poorer sleep quality will be associated with lower psychological resilience, which in turn will be associated with higher loneliness.

H3: Self-esteem will moderate the association between psychological resilience and loneliness. The negative association between psychological resilience and loneliness will be stronger among students with lower self-esteem.

H4: The indirect association between sleep quality and loneliness through psychological resilience will vary by level of self-esteem, indicating a moderated mediation effect.

## 2 Methods

### 2.1 Study design and participants

This cross-sectional study was conducted between May and December 2023 in three higher vocational colleges in Heilongjiang Province, China. A school-based convenience cluster sampling method was used. Students in higher vocational colleges are administratively organized by class, and the survey was conducted during scheduled teaching periods. After permission was obtained from the participating institutions, classes from the first, second, and third academic years were selected as survey units. All eligible students in the selected classes were invited to participate. The questionnaire was administered collectively during class or designated survey periods by trained investigators. A total of 4,000 questionnaires were distributed. After excluding questionnaires with substantial missing information, repeated submissions, obvious response regularity, or logical inconsistency, 3,782 valid questionnaires were included in the final analysis, yielding an effective response rate of 94.55%.

The inclusion criteria were as follows: students enrolled in one of the three participating higher vocational colleges; first-year, second-year, or third-year students; ability to understand the questionnaire content and complete the survey independently; and voluntary participation with informed consent. The exclusion criteria were as follows: refusal to participate; incomplete responses on core study variables; repeated responses; and questionnaires judged invalid according to predefined quality-control criteria. This study was approved by the Ethics Committee of Harbin Medical University (Approval No. HMUIRB20200002) and was conducted in accordance with the Declaration of Helsinki. All participants were informed of the study purpose, voluntary nature of participation, anonymity of responses, and confidentiality of data before completing the questionnaire. Electronic or written informed consent was obtained from all participants prior to the survey.

### 2.2 Measures

#### 2.2.1 Sociodemographic and school-related characteristics

A self-designed general information questionnaire was used to collect sociodemographic, family, school, and lifestyle characteristics. The variables included age, sex, grade, residence, only-child status, family economic status, parental marital status, dormitory relationship, classmate relationship, academic pressure, and physical activity frequency.

#### 2.2.2 Sleep quality

Sleep quality was assessed using the Pittsburgh Sleep Quality Index (PSQI) (Buysse et al., 1989). The PSQI is a self-rated instrument designed to assess sleep quality and sleep disturbances during the previous month. It contains 19 self-rated items that generate seven component scores: subjective sleep quality, sleep latency, sleep duration, habitual sleep efficiency, sleep disturbance, use of sleeping medication, and daytime dysfunction. A sample item is “During the past month, how would you rate your sleep quality overall?” The global PSQI score ranges from 0 to 21, with higher scores indicating poorer sleep quality. In the present study, the Cronbach’s alpha coefficient for the PSQI was 0.831.

#### 2.2.3 Psychological resilience

Psychological resilience was measured using the 10-item Connor-Davidson Resilience Scale (CD-RISC-10) (Campbell-Sills and Stein, 2007). The CD-RISC-10 assesses an individual’s ability to cope with stress, recover from adversity, and maintain adaptive functioning. A sample item is “I am able to adapt when changes occur.” Each item is rated on a 5-point Likert scale ranging from 0 to 4. The total score ranges from 0 to 40, with higher scores indicating greater psychological resilience. In the present study, the Cronbach’s alpha coefficient for the CD-RISC-10 was 0.936.

#### 2.2.4 Self-esteem

Self-esteem was assessed using the 10-item Rosenberg Self-Esteem Scale (RSES) (Rosenberg, 1965). The RSES measures global self-worth and self-acceptance. A sample item is “On the whole, I am satisfied with myself.” Items are rated on a 4-point Likert scale. Negatively worded items were reverse-coded, and higher total scores indicated higher levels of self-esteem. In this study, the Cronbach’s alpha coefficient for the RSES was 0.904.

#### 2.2.5 Loneliness

Loneliness was assessed using the 20-item UCLA Loneliness Scale (Russell, 1996). This scale measures subjective feelings of loneliness and perceived social isolation. A sample item is “How often do you feel that you lack companionship?” Items are rated on a 4-point Likert scale, and negatively worded items were reverse-coded according to the scoring instructions. The total score ranges from 20 to 80, with higher scores indicating greater loneliness. In this study, the Cronbach’s alpha coefficient for the UCLA Loneliness Scale was 0.944.

### 2.3 Quality control

Standardized quality-control procedures were applied before, during, and after data collection. The questionnaire was reviewed before the formal survey to ensure clarity, logical consistency, and suitability for higher vocational college students. Investigators received unified training on the study protocol, questionnaire administration, informed consent procedures, and invalid-response criteria. During the survey, participants were informed that their responses were anonymous and voluntary and that there were no right or wrong answers, reducing response pressure and potential social desirability bias. To avoid duplicate responses, each participant was allowed to submit the questionnaire only once. After data collection, the research team screened all questionnaires and excluded responses with excessive missing data, repeated submissions, obvious patterned responses, or logical contradictions.

### 2.4 Statistical analysis

All statistical analyses were conducted using IBM SPSS Statistics version 26.0 (IBM Corp., Armonk, NY, USA). Confirmatory factor analyses for the assessment of common method bias were performed using IBM SPSS Amos version 26.0. Potential common method bias was assessed using Harman’s single-factor test and confirmatory factor analysis. All observed indicators were entered into an unrotated factor analysis, and a one-factor model was compared with the hypothesized four-factor model (Podsakoff et al., 2003). Descriptive statistics were used to summarize participant characteristics and the distributions of the main variables. Continuous variables were presented as means and standard deviations, and categorical variables were presented as frequencies and percentages. Independent-samples *t* tests were used to compare loneliness scores between two groups, and one-way analysis of variance was used for comparisons among three or more groups. Pearson correlation analysis was used to examine bivariate associations among sleep quality, psychological resilience, self-esteem, and loneliness. Multiple linear regression analyses were conducted to examine the associations among sleep quality, psychological resilience, and loneliness. The mediation model was tested using Model 4 of the PROCESS macro for SPSS, with sleep quality as the independent variable, psychological resilience as the mediator, and loneliness as the dependent variable. The indirect effect was estimated using 5,000 bootstrap resamples and was considered statistically significant when the corresponding 95% bootstrap confidence interval did not include zero (Igartua and Hayes, 2021).

To test the moderated mediation model, psychological resilience and self-esteem were mean-centered before calculating the interaction term. The moderated mediation model was tested using Model 14 of the PROCESS macro for SPSS (Igartua and Hayes, 2021), in which self-esteem was specified as a moderator of the association between psychological resilience and loneliness. Conditional indirect effects were estimated at low, mean, and high levels of self-esteem. The index of moderated mediation was calculated to determine whether the indirect association between sleep quality and loneliness through psychological resilience varied by self-esteem level. All fully adjusted models included age, sex, grade, residence, family economic status, parental marital status, dormitory relationship, classmate relationship, academic pressure, and physical activity frequency. Age was entered as a continuous variable, and ordered categorical variables were entered according to their ordinal levels where applicable. Statistical significance was defined as a two-sided *p* value <0.05.

## 3 Results

### 3.1 Participant characteristics

A total of 3,782 students were included in the final analysis, including 1,593 males (42.1%) and 2,189 females (57.9%). By academic year, 34.7% of participants were in first year, 35.1% in second year, and 30.2% in third year. The mean loneliness score was 39.48 ± 7.75. As shown in Table 1, loneliness scores differed significantly by age group, sex, grade, residence, family economic status, parental marital status, dormitory relationship, classmate relationship, academic pressure, and physical activity frequency (all *p* < 0.05). Only-child status was not significantly associated with loneliness (*p* = 0.212). In general, higher loneliness scores were observed among older students, female students, third-year students, rural students, students whose parents were divorced, separated, or widowed, those reporting poorer dormitory or classmate relationships, those with higher academic pressure, and those who rarely or never participated in physical activity.

**Table 1:** Participants’ characteristics and the distribution of loneliness.

Variables	*N* (%)	Loneliness, Mean ± SD	*F/t*	*p*
Age group				
≤18	1,038 (27.4)	39.11 ± 7.36	4.452	0.012
19–20	2044 (54.0)	39.38 ± 7.54		
≥21	700 (18.5)	40.18 ± 7.80		
Sex				
Male	1,593 (42.1)	38.96 ± 7.43	−3.430	<0.001
Female	2,189 (57.9)	39.81 ± 7.61		
Grade				
First-year	1,314 (34.7)	38.75 ± 7.28	9.651	<0.001
Second-year	1,326 (35.1)	39.65 ± 7.70		
Third-year	1,142 (30.2)	40.04 ± 7.61		
Residence				
Urban	1732 (45.8)	39.07 ± 7.79	−2.876	0.004
Rural	2050 (54.2)	39.78 ± 7.32		
Only child				
Yes	1,014 (26.8)	39.20 ± 7.54	−1.249	0.212
No	2,768 (73.2)	39.55 ± 7.55		
Family economic status				
Low	794 (21.0)	39.19 ± 7.50	24.552	<0.001
Middle	2,530 (66.9)	41.00 ± 7.82		
High	458 (12.1)	38.22 ± 6.95		
Parental marital status				
Married/cohabiting	3,342 (88.4)	39.36 ± 7.75	−2.808	0.005
Divorced/separated/widowed	440 (11.6)	40.45 ± 7.68		
Dormitory relationship				
Good	1891 (50.0)	38.99 ± 7.41	9.423	<0.001
Moderate	1,399 (37.0)	39.70 ± 7.69		
Poor	492 (13.0)	40.54 ± 7.52		
Classmate relationship				
Good	2080 (55.0)	39.07 ± 7.47	7.703	<0.001
Moderate	1,323 (35.0)	39.75 ± 7.70		
Poor	379 (10.0)	40.54 ± 7.30		
Academic pressure				
Low	757 (20.0)	38.81 ± 7.41	7.422	<0.001
Moderate	2,117 (56.0)	39.36 ± 7.55		
High	908 (24.0)	40.20 ± 7.60		
Physical activity frequency				
≥3 times/week	946 (25.0)	38.83 ± 7.52	7.175	<0.001
1–2 times/week	1701 (45.0)	39.39 ± 7.33		
Rarely/Never	1,135 (30.0)	40.07 ± 7.84		

*t* tests were used for two-group comparisons and one-way ANOVA was used for variables with three groups. *p* < 0.05 was considered statistically significant.

### 3.2 Common method bias assessment

Because the main variables were measured using self-report questionnaires, common method bias was examined. Harman’s single-factor test showed that four factors had eigenvalues greater than 1, and the first factor accounted for 34.33% of the total variance. This result indicated that no single factor dominated the covariance among the study variables. In addition, confirmatory factor analysis showed that the one-factor model fitted poorly, whereas the theoretical four-factor model consisting of sleep quality, psychological resilience, self-esteem, and loneliness showed substantially better model fit. Taken together, these findings indicated that the observed relationships were unlikely to be explained primarily by a single common method factor. Detailed results are presented in Supplementary Table S1.

### 3.3 Descriptive statistics and correlations among the main variables

Table 2 presents the descriptive statistics and correlations among the main variables. The mean scores were 7.21 ± 2.75 for sleep quality, 25.09 ± 5.33 for psychological resilience, 26.10 ± 4.40 for self-esteem, and 39.48 ± 7.75 for loneliness. Pearson correlation analysis showed that sleep quality was positively correlated with loneliness (*r* = 0.580, *p* < 0.01), indicating that poorer sleep quality was associated with higher loneliness. Sleep quality was negatively correlated with psychological resilience (*r* = −0.516, *p* < 0.01) and self-esteem (*r* = −0.330, *p* < 0.01). Psychological resilience was positively correlated with self-esteem (*r* = 0.505, *p* < 0.01) and negatively correlated with loneliness (*r* = −0.682, *p* < 0.01). Self-esteem was also negatively correlated with loneliness (*r* = −0.566, *p* < 0.01).

**Table 2:** Description statistics and correlation analysis of each variable.

Variable	M	SD	1	2	3	4	5
Age	19.35	1.31	1				
Sleep quality	7.21	2.75	0.064**	1			
Psychological resilience	25.09	5.33	−0.019	−0.516**	1		
Self-esteem	26.10	4.40	−0.036*	−0.330**	0.505**	1	
Loneliness	39.48	7.75	0.046**	0.580**	−0.682**	−0.566**	1

Correlations are Pearson correlation coefficients. ^*^p < 0.05, ^**^p < 0.01.

### 3.4 Mediating role of psychological resilience in the association between sleep quality and loneliness

Multiple linear regression analyses showed that sleep quality was significantly associated with loneliness after adjustment for age, sex, grade, residence, family economic status, parental marital status, dormitory relationship, classmate relationship, academic pressure, and physical activity frequency. As shown in Table 3, poorer sleep quality was significantly associated with higher loneliness in the total-effect model (*B* = 1.527, SE = 0.038, *β* = 0.556, *p* < 0.01). Poorer sleep quality was also significantly associated with lower psychological resilience (*B* = −0.951, SE = 0.028, *β* = −0.491, *p* < 0.01). When sleep quality and psychological resilience were entered simultaneously, psychological resilience was negatively associated with loneliness (*B* = −0.738, SE = 0.019, *β* = −0.521, *p* < 0.01), while sleep quality remained positively associated with loneliness (direct effect: *B* = 0.825, SE = 0.037, *β* = 0.300, *p* < 0.01).

**Table 3:** Regression analyses for the mediation model.

Regression equation		Overall fit coefficient			Regression coefficient				
Outcome variables	Predictor variables	*R*	*R* ^2^	*F*	*β*	*B*	*SE*	*t*	95%CI
Loneliness	Sleep quality	0.571	0.326	165.850**	0.556	1.527	0.038	40.155**	(1.453, 1.602)
Psychological resilience	Sleep quality	0.529	0.280	132.959**	−0.491	−0.951	0.028	−34.268**	(−1.006, −0.896)
Loneliness	Sleep quality	0.722	0.522	342.541**	0.300	0.825	0.037	22.483**	(0.752, 0.898)
	Psychological resilience				−0.521	−0.738	0.019	−39.255**	(−0.775, −0.701)

^*^p < 0.05, ^**^p < 0.01. Only the key predictors in the mediation paths are shown. All models were adjusted for age, sex, grade, residence, family economic status, parental marital status, dormitory relationship, classmate relationship, academic pressure, and physical activity frequency. Ordered categorical variables were entered according to their ordinal levels where applicable.

Bootstrap analysis further showed a significant indirect effect of sleep quality on loneliness through psychological resilience (indirect effect = 0.701, SE = 0.027, 95% CI: 0.649 to 0.755), accounting for 45.93% of the total effect (Table 4). The direct effect also remained significant (direct effect = 0.826, SE = 0.036, 95% CI: 0.755 to 0.894), supporting a partial mediating role of psychological resilience. Full adjusted regression results are provided in Supplementary Table S2.

**Table 4:** Total, direct, and indirect effects of sleep quality on loneliness through psychological resilience.

Path	*B*	SE	95% CI	Ratio of effect values
Total effect	1.527	0.038	(1.453, 1.602)	
Direct effect	0.826	0.036	(0.755, 0.894)	54.07%
Indirect effect	0.701	0.027	(0.649, 0.755)	45.93%

Bootstrap estimates were based on 5,000 resamples. CI, confidence interval.

### 3.5 Moderated mediation analysis

**Figure 1:**
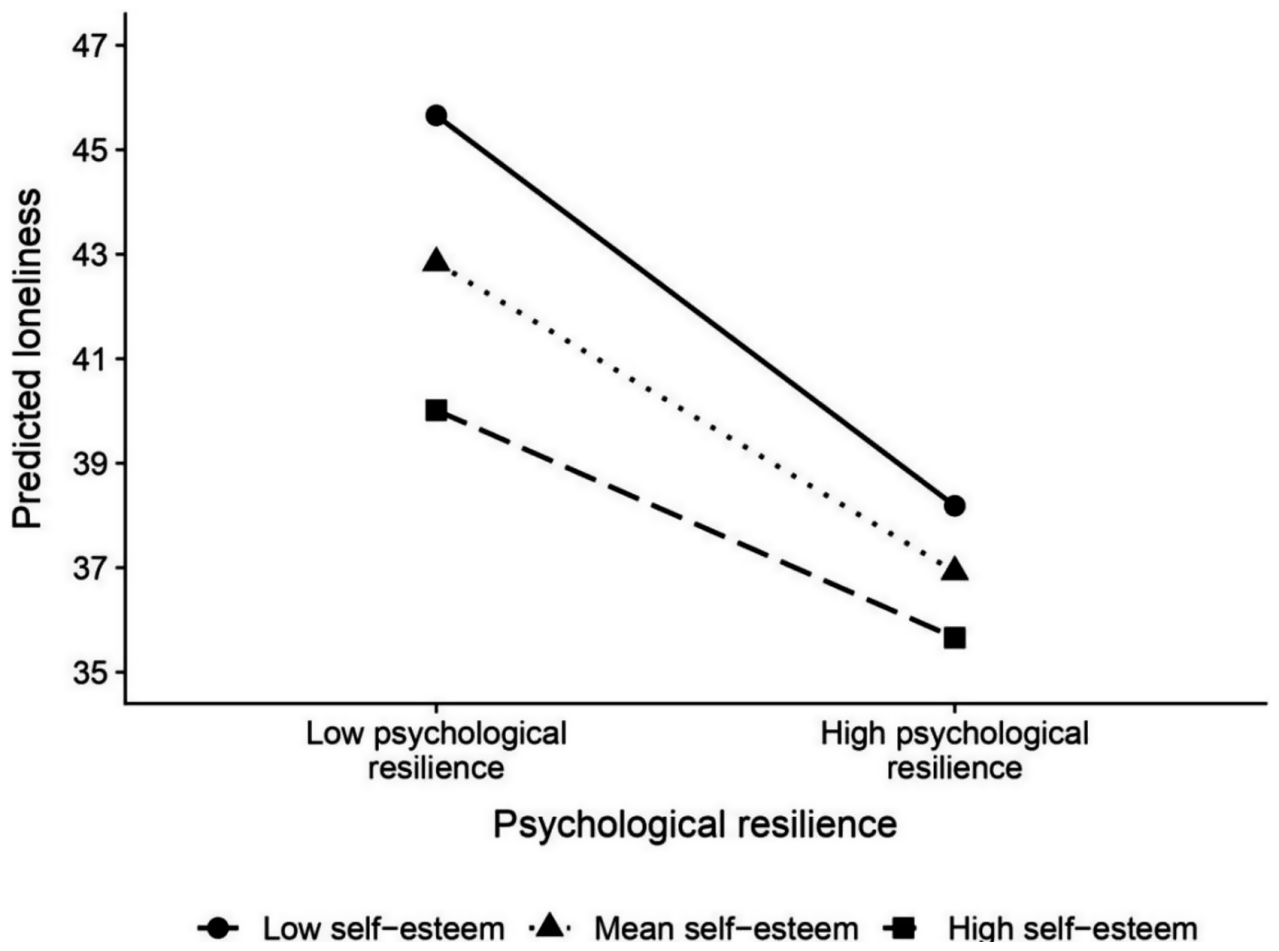
Moderating effect of self-esteem on the association between psychological resilience and loneliness. Predicted loneliness was estimated at low and high levels of psychological resilience and at low, mean, and high levels of self-esteem. The model was adjusted for age, sex, grade, residence, family economic status, parental marital status, dormitory relationship, classmate relationship, academic pressure, and physical activity frequency.

The moderated mediation model further examined whether self-esteem moderated the pathway from psychological resilience to loneliness. After adjustment for sleep quality, psychological resilience, self-esteem, and covariates, the interaction between psychological resilience and self-esteem was statistically significant (*B* = 0.035, SE = 0.003, *β* = 0.119, *p* < 0.01), indicating that the association between psychological resilience and loneliness varied by level of self-esteem. As shown in Figure 1, psychological resilience was negatively associated with loneliness across different levels of self-esteem, but this negative association was stronger among students with lower self-esteem and weaker among those with higher self-esteem.

**Figure 2:**
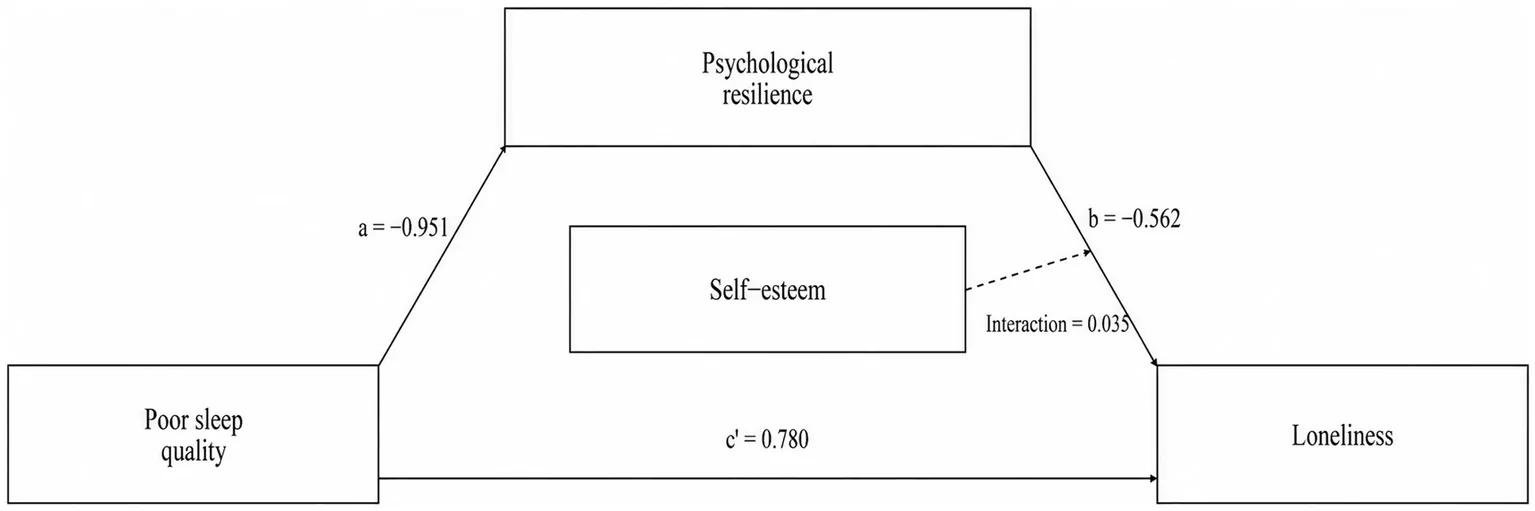
Conceptual framework linking sleep quality, psychological resilience, self-esteem, and loneliness. Coefficients are unstandardized regression estimates. Higher PSQI scores indicate poorer sleep quality. The model was adjusted for age, sex, grade, residence, family economic status, parental marital status, dormitory relationship, classmate relationship, academic pressure, and physical activity frequency. Full regression results are presented in Table 6, Supplementary Table S3, and conditional indirect effects are presented in Table 5.

Conditional indirect effect analysis showed that the indirect effect of sleep quality on loneliness through psychological resilience varied across levels of self-esteem, consistent with the moderated mediation framework shown in Figure 2. The indirect effect was strongest among students with low self-esteem (estimate = 0.662, Boot SE = 0.034, 95% CI: 0.622 to 0.738), moderate among those with average self-esteem (estimate = 0.524, Boot SE = 0.028, 95% CI: 0.489–0.580), and weakest among those with high self-esteem (estimate = 0.386, Boot SE = 0.029, 95% CI: 0.344 to 0.436). The index of moderated mediation was significant (index = −0.031, Boot SE = 0.003, 95% CI: −0.039 to −0.027), confirming that the indirect association between sleep quality and loneliness through psychological resilience decreased as self-esteem increased (Table 5). The regression results for the interaction between psychological resilience and self-esteem are shown in Table 6, and the fully adjusted regression results are provided in Supplementary Table S3.

**Table 5:** Conditional indirect effects and index of moderated mediation.

Effect type	Moderator level	Estimate	Boot SE	Boot LLCI	Boot ULCI
Conditional indirect effect	Low self-esteem (−1 SD, 21.50)	0.662	0.034	0.622	0.738
Conditional indirect effect	Average self-esteem (M, 25.93)	0.524	0.028	0.489	0.580
Conditional indirect effect	High self-esteem (+1 SD, 30.36)	0.386	0.029	0.344	0.436
Index of moderated mediation	Self-esteem	−0.031	0.003	−0.039	−0.027

Boot LLCI and Boot ULCI indicate the lower and upper limits of the 95% bootstrap confidence interval. Bootstrap estimates were based on 5,000 resamples.

**Table 6:** Regression analyses for the moderated mediation model.

Regression equation		Overall fit coefficient			Regression coefficient				
Outcome variables	Predictor variables	*R*	*R* ^2^	*F*	*β*	*B*	SE	*t*	95%*CI*
Psychological resilience	Sleep quality	0.529	0.280	132.959**	−0.491	−0.951	0.028	−34.268**	(−1.006, −0.896)
Loneliness	Sleep quality	0.767	0.588	384.207**	0.284	0.780	0.034	22.830**	(0.713, 0.847)
	Psychological resilience				−0.397	−0.562	0.019	−29.438**	(−0.599, −0.525)
	Self-esteem				−0.269	−0.461	0.021	−21.945**	(−0.502, −0.420)
	Psychological resilience × self-esteem				0.119	0.035	0.003	11.348**	(0.029, 0.041)

^*^p < 0.05, ^**^p < 0.01. Only the key predictors and the interaction term are shown. Models were adjusted for age, sex, grade, residence, family economic status, parental marital status, dormitory relationship, classmate relationship, academic pressure, and physical activity frequency. Psychological resilience and self-esteem were mean-centered before calculating the interaction term.

### 3.6 Subgroup analysis

**Figure 3:**
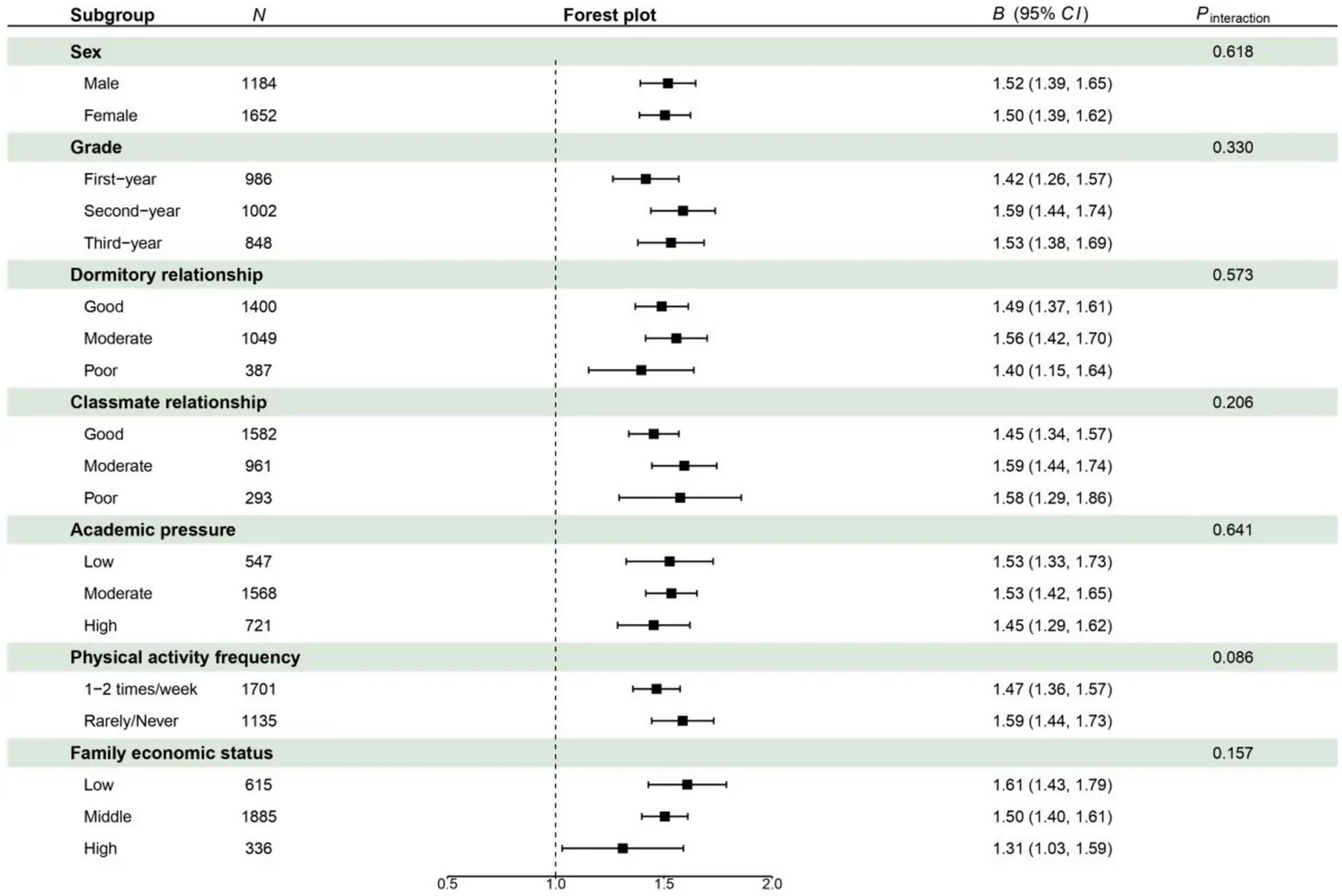
Subgroup analysis of the association between sleep quality and loneliness.

Subgroup analyses were conducted to examine whether the association between sleep quality and loneliness was consistent across different participant groups. As shown in Figure 3, poorer sleep quality was positively associated with loneliness across subgroups stratified by sex, grade, dormitory relationship, classmate relationship, academic pressure, physical activity frequency, and family economic status. The tests for interaction were not statistically significant across these subgroup variables, suggesting that the positive association between sleep quality and loneliness was generally robust across different student subgroups.

## 4 Discussion

This study examined the association between sleep quality and loneliness among higher vocational college students and further tested the mediating role of psychological resilience and the moderating role of self-esteem. The results showed that poorer sleep quality was significantly associated with higher loneliness after adjustment for sociodemographic, family, school relationship, academic pressure, and physical activity variables. Psychological resilience partially mediated this association, accounting for 45.93% of the total effect. Self-esteem further moderated the pathway from psychological resilience to loneliness, with the indirect association between sleep quality and loneliness through psychological resilience being stronger among students with lower self-esteem and weaker among those with higher self-esteem. These findings suggest that loneliness among higher vocational college students is not only related to interpersonal or social conditions, but also to everyday health related factors and internal psychological resources. The results are consistent with the growing literature that conceptualizes loneliness as a salient issue among young people and emerging adults, especially during periods of educational, residential, and interpersonal transition (Kirwan et al., 2025).

The present study found that poorer sleep quality was positively associated with loneliness. This finding is consistent with a systematic review and meta-analysis showing that loneliness is related to self reported sleep disturbance, although the longitudinal direction between loneliness and sleep remains uncertain (Deng et al., 2023). Recent evidence among university students has also shown that loneliness is closely associated with poor sleep quality. A sex-specific analysis further suggested that loneliness may be involved in the psychological pathways linking bedtime procrastination and sleep quality among male and female students (Wang et al., 2025b). The present study adds to this literature by examining the sleep quality and loneliness relationship in higher vocational college students, a group that has received less empirical attention than students in general universities.

Several mechanisms may explain the observed association. Poor sleep quality may reduce emotional regulation capacity, increase fatigue and irritability, and heighten sensitivity to negative social cues. Students with poor sleep may therefore find it more difficult to maintain stable peer interactions, manage dormitory conflicts, or interpret ambiguous interpersonal situations in a balanced manner. Previous research on young people has suggested that loneliness is linked with negative interpretations of social situations, which may contribute to the maintenance of loneliness (Kharbush et al., 2026). In this context, poor sleep quality may act as an everyday vulnerability factor that makes students more susceptible to loneliness. Importantly, sleep quality is also potentially modifiable. A recent systematic review and meta-analysis found that psychological interventions can improve sleep quality among university students, indicating that sleep problems may be amenable to intervention in educational settings (Tadros et al., 2025).

Psychological resilience partially mediated the association between sleep quality and loneliness. Specifically, poorer sleep quality was associated with lower psychological resilience, which in turn was associated with higher loneliness. This finding is theoretically plausible. Resilience reflects the capacity to adapt to stress, recover from adversity, and maintain psychological functioning under challenging circumstances. According to Conservation of Resources Theory, individuals seek to preserve and restore psychological resources, and repeated stress or inadequate recovery may deplete these resources (Li and Hahn, 2026). Poor sleep quality may undermine students’ psychological recovery and interpersonal functioning, leaving them with fewer resources to cope with academic pressure, peer friction, and residential adjustment. Recent studies among university students have shown that poorer sleep quality is associated with higher psychological distress and lower interpersonal functioning, supporting the role of sleep as an everyday condition linked to students’ emotional and social adjustment (Farrell et al., 2024).

Recent empirical evidence supports this interpretation. Studies among college students have indicated that sleep quality is closely associated with psychological resilience, and that poor sleep may be related to lower resilience or higher psychological vulnerability (Li and Guo, 2023). Recent evidence also suggests that sleep quality and resilience may jointly explain associations with mental health symptoms, supporting the view that resilience is a relevant psychological pathway linking sleep-related difficulties to student mental health (Poon et al., 2024). The present study extends this evidence to loneliness. Rather than viewing loneliness only as a consequence of insufficient social contact, the findings suggest that reduced adaptive capacity may partly explain why students with poorer sleep quality experience greater loneliness (Wang et al., 2025a). The indirect effect through psychological resilience accounted for 45.93% of the total association, indicating that resilience represented a substantively relevant component of the observed relationship. However, more than half of the association remained unexplained, suggesting that other emotional and interpersonal processes, such as negative affect, social withdrawal, interpersonal sensitivity, and perceived social support, may also be involved.

The size of the indirect effect also deserves attention. Psychological resilience accounted for nearly half of the total association between sleep quality and loneliness. This suggests that resilience is not merely a peripheral variable, but a central psychological resource in the sleep and loneliness pathway. Previous research among college students indicates that loneliness is closely related to social support, shared activities, and psychological resources, suggesting that both interpersonal and individual adaptive factors should be considered when explaining students’ loneliness (Cipolletta et al., 2025). Recent studies in college student populations further suggest that positive psychological resources, including self-esteem and resilience, are relevant to student well-being and may inform mental health promotion strategies (Xie et al., 2025). The present findings are consistent with this perspective and indicate that resilience may be a key target for reducing loneliness among students with poor sleep quality.

The present study further found that self-esteem moderated the association between psychological resilience and loneliness. The negative association between psychological resilience and loneliness was stronger among students with lower self-esteem and weaker among those with higher self-esteem. In the present study, the conditional indirect effect of sleep quality on loneliness through psychological resilience decreased as self-esteem increased. This pattern suggests that self-esteem may function as a protective self-evaluative resource, weakening the association between reduced resilience and loneliness (Ismailova et al., 2025).

This finding is consistent with Sociometer theory, which proposes that self-esteem functions as an interpersonal monitor of perceived relational value and possible social exclusion (Leary et al., 1995). Students with higher self-esteem may be more likely to maintain a stable sense of self-worth when facing stress or interpersonal difficulties. In contrast, students with lower self-esteem may be more likely to interpret setbacks, dormitory conflicts, or peer difficulties as evidence of rejection or inadequacy. Under such conditions, reduced psychological resilience may be more readily translated into loneliness. Previous research among young students has shown that higher loneliness is associated with lower self-esteem and poorer psychological well-being (Hernández-Díaz et al., 2025). Other studies have examined self-esteem as a moderator in the association between loneliness and well-being-related outcomes, supporting its potential role as a boundary condition in loneliness-related psychological adjustment (Moksnes et al., 2022).

The moderated mediation finding has important theoretical implications. It suggests that psychological resilience and self-esteem should not be treated as interchangeable positive psychological resources. Resilience appears to explain part of the pathway from sleep quality to loneliness, whereas self-esteem appears to shape the strength of the resilience–loneliness association. In other words, resilience may represent adaptive coping capacity, while self-esteem may represent a self-evaluative resource related to perceived relational value and social acceptance. The gradual reduction in the conditional indirect effect across increasing levels of self-esteem further suggests that higher self-esteem may provide partial protection when resilience is limited, although it does not eliminate the association between sleep quality and loneliness. This distinction helps clarify why students with similar levels of sleep difficulty may differ in loneliness outcomes.

The findings carry several implications for loneliness reduction among higher vocational college students. Sleep quality deserves greater attention in both student loneliness research and school-based mental health practice. Existing approaches to loneliness often emphasize social skills, peer interaction, and social support. These approaches remain important, but the present findings suggest that everyday health-related conditions, particularly poor sleep quality, may also be relevant to students’ loneliness. Sleep education, regular sleep schedules, dormitory sleep environment management, and early identification of sleep problems may therefore provide practical entry points for student support programs. Psychological resilience may represent another important target for loneliness prevention. Programs that strengthen emotion regulation, coping flexibility, stress management, and problem solving may help students with poor sleep quality maintain adaptive functioning and reduce their vulnerability to loneliness. This is particularly relevant for higher vocational college students, who may face concurrent demands related to academic adaptation, skill training, dormitory life, and employment preparation. Such programs could be incorporated into routine mental health education or delivered through brief group-based activities for students reporting persistent sleep difficulties or elevated loneliness. Self-esteem may also help identify students who are more vulnerable to loneliness. The conditional indirect effect was strongest among students with lower self-esteem, suggesting that sleep problems and reduced resilience may be more closely linked to loneliness in this subgroup. School-based mental health services may therefore consider integrating sleep health education, resilience-building activities, and self-esteem support into routine student programs. Sleep-related components could address regular sleep schedules, healthy bedtime routines, nighttime digital-media use, dormitory sleep environments, and early recognition of persistent sleep problems. Resilience-building activities could focus on emotional regulation, coping flexibility, stress management, and interpersonal problem solving, while self-esteem support could emphasize self-acceptance, perceived competence, and balanced interpretations of interpersonal experiences. These approaches may help students manage sleep-related and psychological vulnerabilities associated with loneliness.

## 5 Strengths, limitations, and recommendations

This study has several strengths. The relatively large sample of higher vocational college students from three schools provides useful evidence for a population that remains underrepresented in loneliness research. The study also integrated sleep quality, psychological resilience, self-esteem, and loneliness within a single conditional process model, allowing both mediating and moderating mechanisms to be examined simultaneously. In addition, the analyses adjusted for a range of sociodemographic, family, school-related, academic pressure, and physical activity variables, which strengthened the robustness of the observed associations. Methodologically, common method bias was assessed using both Harman’s single-factor test and confirmatory factor analysis, and the results did not suggest serious common method bias.

Several limitations should be acknowledged. The cross-sectional design prevents causal inference, even though the proposed model was theoretically grounded. The temporal ordering among sleep quality, psychological resilience, self-esteem, and loneliness therefore cannot be established, and longitudinal or intervention studies are needed to clarify directionality. All variables were measured using self-report scales, which may have introduced response bias, including recall error and socially desirable responding. Future studies could incorporate objective sleep indicators, ecological momentary assessment, or multi-informant data to strengthen measurement validity. Residual confounding is also possible. Although the models adjusted for multiple covariates, depressive symptoms, anxiety symptoms, perceived social support, and problematic smartphone use were not assessed and may have influenced the observed associations. Future studies should incorporate these factors when examining the relationships among sleep quality, psychological resources, and loneliness. The sample was recruited from three higher vocational colleges in Heilongjiang Province, which may limit the generalizability of the findings. The observed associations may not apply directly to students from other regions of China, traditional universities, different vocational education systems, or other cultural settings. Future multicenter longitudinal studies involving more geographically, educationally, and culturally diverse student populations are needed to examine the stability and temporal direction of these associations and to evaluate interventions targeting sleep quality, psychological resilience, and self-esteem.

## 6 Conclusion

This study showed that poorer sleep quality was associated with greater loneliness among higher vocational college students. Psychological resilience partially mediated this association, while self-esteem moderated the link between resilience and loneliness. The findings indicate that loneliness in this population is related not only to interpersonal experiences but also to sleep-related vulnerability and internal psychological resources. Students with poor sleep, low resilience, and low self-esteem may warrant particular attention. Higher vocational colleges should consider incorporating sleep health education, resilience-building activities, and self-esteem support into routine student mental health services. Strengthening sleep management, adaptive coping, emotional regulation, and positive self-evaluation may help reduce vulnerability to loneliness and improve students’ psychological adjustment and social functioning.

## Data Availability

The datasets generated and analyzed during the current study are not publicly available due to participant privacy and ethical restrictions. De-identified data may be made available from the corresponding author upon reasonable request, subject to approval by the relevant ethics committee and participating institutions.
