# Supplementary material for: Poor sleep quality and loneliness among higher vocational college students: a conditional process analysis of psychological resilience and self-esteem
Source: Front Psychol. 2026 Aug 21;17:1893857. doi: 10.3389/fpsyg.2026.1893857 (PMC13541442; doi:10.3389/fpsyg.2026.1893857)
Supplement: Supplementary file 1 [file Supplementary_file_1.DOCX]

**Supplementary Tables for Fully Adjusted Models**

The fully adjusted models included all variables that were significant in the univariate comparisons with loneliness: age, sex, grade, residence, family economic status, parental marital status, dormitory relationship, classmate relationship, academic pressure, and physical activity frequency. Age was entered as a continuous variable. Only child status was not included because it was not statistically significant in the univariate analysis.

**Supplementary Table S1. Results of common method bias assessment.**

| **Test** | **Analytic strategy** | **Key result** | **Fit indices or criteria** | **Interpretation** |
| --- | --- | --- | --- | --- |
| Harman single factor test | All 47 item level indicators were entered into an unrotated exploratory factor analysis. | Four factors had eigenvalues greater than 1. The first factor explained 34.33% of the total variance. | First factor variance < 40%. | No single factor accounted for the majority of item covariance. |
| One factor CFA | All 47 items were specified to load on one latent factor. | Chi square(1034) = 21168.58, p < 0.001; chi square/df = 20.47. | CFI = 0.750; TLI = 0.738; RMSEA = 0.072; SRMR = 0.074. | The one factor model fitted poorly, suggesting that a single common method factor did not adequately represent the data. |
| Four factor CFA comparison | Items were specified according to the theoretical constructs: sleep quality, psychological resilience, self-esteem, and loneliness. | Chi square(1028) = 1084.81, p = 0.107; chi square/df = 1.06. | CFI = 0.999; TLI = 0.999; RMSEA = 0.004; SRMR = 0.012. | The theoretical four factor model fitted substantially better than the one factor model, supporting construct distinction. |

Note. CFA, confirmatory factor analysis; CFI, comparative fit index; TLI, Tucker Lewis index; RMSEA, root mean square error of approximation; SRMR, standardized root mean square residual.

**Supplementary Table S2. Fully adjusted hierarchical regression results for the mediation model.**

| **Regression equation** | | **Overall fit coefficient** | | | **Regression coefficient** | | | |
| --- | --- | --- | --- | --- | --- | --- | --- | --- |
| **Outcome variables** | **Predictor variables** | **R** | **R²** | **F** | **β** | **B** | **SE** | **t** |
| Loneliness | Sleep quality | 0.571 | 0.326 | 165.850** | 0.556 | 1.527 | 0.038 | 40.155** |
|  | Age |  |  |  | -0.000 | -0.001 | 0.112 | -0.009 |
|  | Sex |  |  |  | 0.038 | 0.584 | 0.205 | 2.853** |
|  | Grade |  |  |  | 0.017 | 0.157 | 0.183 | 0.862 |
|  | Residence |  |  |  | 0.033 | 0.498 | 0.203 | 2.455* |
|  | Family economic status |  |  |  | -0.063 | -0.838 | 0.178 | -4.699** |
|  | Parental marital status |  |  |  | 0.031 | 0.728 | 0.315 | 2.309* |
|  | Dormitory relationship |  |  |  | 0.001 | 0.009 | 0.145 | 0.062 |
|  | Classmate relationship |  |  |  | 0.014 | 0.153 | 0.152 | 1.007 |
|  | Academic pressure |  |  |  | -0.016 | -0.188 | 0.154 | -1.219 |
|  | Physical activity frequency |  |  |  | -0.007 | -0.071 | 0.138 | -0.519 |
| Psychological resilience | Sleep quality | 0.529 | 0.280 | 132.959** | -0.491 | -0.951 | 0.028 | -34.268** |
|  | Age |  |  |  | 0.009 | 0.035 | 0.082 | 0.426 |
|  | Sex |  |  |  | -0.017 | -0.186 | 0.149 | -1.244 |
|  | Grade |  |  |  | 0.006 | 0.038 | 0.133 | 0.289 |
|  | Residence |  |  |  | -0.008 | -0.082 | 0.148 | -0.553 |
|  | Family economic status |  |  |  | 0.032 | 0.295 | 0.130 | 2.269* |
|  | Parental marital status |  |  |  | -0.004 | -0.071 | 0.230 | -0.309 |
|  | Dormitory relationship |  |  |  | -0.028 | -0.215 | 0.106 | -2.031* |
|  | Classmate relationship |  |  |  | -0.071 | -0.565 | 0.111 | -5.110** |
|  | Academic pressure |  |  |  | -0.051 | -0.411 | 0.112 | -3.658** |
|  | Physical activity frequency |  |  |  | -0.054 | -0.388 | 0.101 | -3.859** |
| Loneliness | Sleep quality | 0.722 | 0.522 | 342.541** | 0.300 | 0.825 | 0.037 | 22.483** |
|  | Psychological resilience |  |  |  | -0.521 | -0.738 | 0.019 | -39.255** |
|  | Age |  |  |  | 0.004 | 0.025 | 0.094 | 0.262 |
|  | Sex |  |  |  | 0.029 | 0.447 | 0.172 | 2.590** |
|  | Grade |  |  |  | 0.020 | 0.186 | 0.154 | 1.207 |
|  | Residence |  |  |  | 0.029 | 0.437 | 0.171 | 2.560* |
|  | Family economic status |  |  |  | -0.047 | -0.620 | 0.150 | -4.123** |
|  | Parental marital status |  |  |  | 0.029 | 0.675 | 0.266 | 2.542* |
|  | Dormitory relationship |  |  |  | -0.014 | -0.149 | 0.122 | -1.224 |
|  | Classmate relationship |  |  |  | -0.023 | -0.265 | 0.128 | -2.065* |
|  | Academic pressure |  |  |  | -0.043 | -0.491 | 0.130 | -3.779** |
|  | Physical activity frequency |  |  |  | -0.035 | -0.358 | 0.116 | -3.078** |

**Note.** **P* < 0.05, ***P* < 0.01. N = 3,782. Age was added to the fully adjusted model and entered as a continuous variable. The covariates were age, sex, grade, residence, family economic status, parental marital status, dormitory relationship, classmate relationship, academic pressure, and physical activity frequency. Ordered categorical variables were entered according to their ordinal levels where applicable.

**Supplementary Table S3. Fully adjusted hierarchical regression results for the moderated mediation model.**

| **Regression equation** | | **Overall fit coefficient** | | | **Regression coefficient** | | | |
| --- | --- | --- | --- | --- | --- | --- | --- | --- |
| **Outcome variables** | **Predictor variables** | **R** | **R²** | **F** | **β** | **B** | **SE** | **t** |
| Psychological resilience | Sleep quality | 0.529 | 0.280 | 132.959** | -0.491 | -0.951 | 0.028 | -34.268** |
|  | Age |  |  |  | 0.009 | 0.035 | 0.082 | 0.426 |
|  | Sex |  |  |  | -0.017 | -0.186 | 0.149 | -1.244 |
|  | Grade |  |  |  | 0.006 | 0.038 | 0.133 | 0.289 |
|  | Residence |  |  |  | -0.008 | -0.082 | 0.148 | -0.553 |
|  | Family economic status |  |  |  | 0.032 | 0.295 | 0.130 | 2.269* |
|  | Parental marital status |  |  |  | -0.004 | -0.071 | 0.230 | -0.309 |
|  | Dormitory relationship |  |  |  | -0.028 | -0.215 | 0.106 | -2.031* |
|  | Classmate relationship |  |  |  | -0.071 | -0.565 | 0.111 | -5.110** |
|  | Academic pressure |  |  |  | -0.051 | -0.411 | 0.112 | -3.658** |
|  | Physical activity frequency |  |  |  | -0.054 | -0.388 | 0.101 | -3.859** |
| Loneliness | Sleep quality | 0.767 | 0.588 | 384.207** | 0.284 | 0.780 | 0.034 | 22.830** |
|  | Psychological resilience |  |  |  | -0.397 | -0.562 | 0.019 | -29.438** |
|  | Self-esteem |  |  |  | -0.269 | -0.461 | 0.021 | -21.945** |
|  | Psychological resilience × self-esteem |  |  |  | 0.119 | 0.035 | 0.003 | 11.348** |
|  | Age |  |  |  | -0.004 | -0.025 | 0.088 | -0.282 |
|  | Sex |  |  |  | 0.025 | 0.379 | 0.160 | 2.369* |
|  | Grade |  |  |  | 0.017 | 0.162 | 0.143 | 1.136 |
|  | Residence |  |  |  | 0.034 | 0.522 | 0.159 | 3.291** |
|  | Family economic status |  |  |  | -0.030 | -0.393 | 0.140 | -2.810** |
|  | Parental marital status |  |  |  | 0.025 | 0.592 | 0.247 | 2.400* |
|  | Dormitory relationship |  |  |  | -0.029 | -0.317 | 0.114 | -2.787** |
|  | Classmate relationship |  |  |  | -0.039 | -0.438 | 0.119 | -3.673** |
|  | Academic pressure |  |  |  | -0.047 | -0.538 | 0.121 | -4.460** |
|  | Physical activity frequency |  |  |  | -0.043 | -0.438 | 0.108 | -4.058** |

**Note.** **P* < 0.05, ***P* < 0.01. N = 3,782. Age was added to the fully adjusted model and entered as a continuous variable. Psychological resilience and self-esteem were mean centered before calculating the interaction term. The covariates were age, sex, grade, residence, family economic status, parental marital status, dormitory relationship, classmate relationship, academic pressure, and physical activity frequency.
